# ^68^Ga DOTATATE Uptake in Hemangioma Simulating Metastasis on PET Imaging: CT helps characterize bone hemangioma that could be wrongly interpreted as skeletal metastases on ^68^Ga DOTATATE PET imaging

**DOI:** 10.5334/jbsr.1772

**Published:** 2019-06-28

**Authors:** Bart Vertenten, Lode Goethals, Frank De Geeter

**Affiliations:** 1 UZ Brussel, BE; 2 AZ Sint-Jan Brugge-Oostende, BE

**Keywords:** ^68^Ga DOTATATE, vertebral hemangioma, PET

## Case

**Figure 1:**
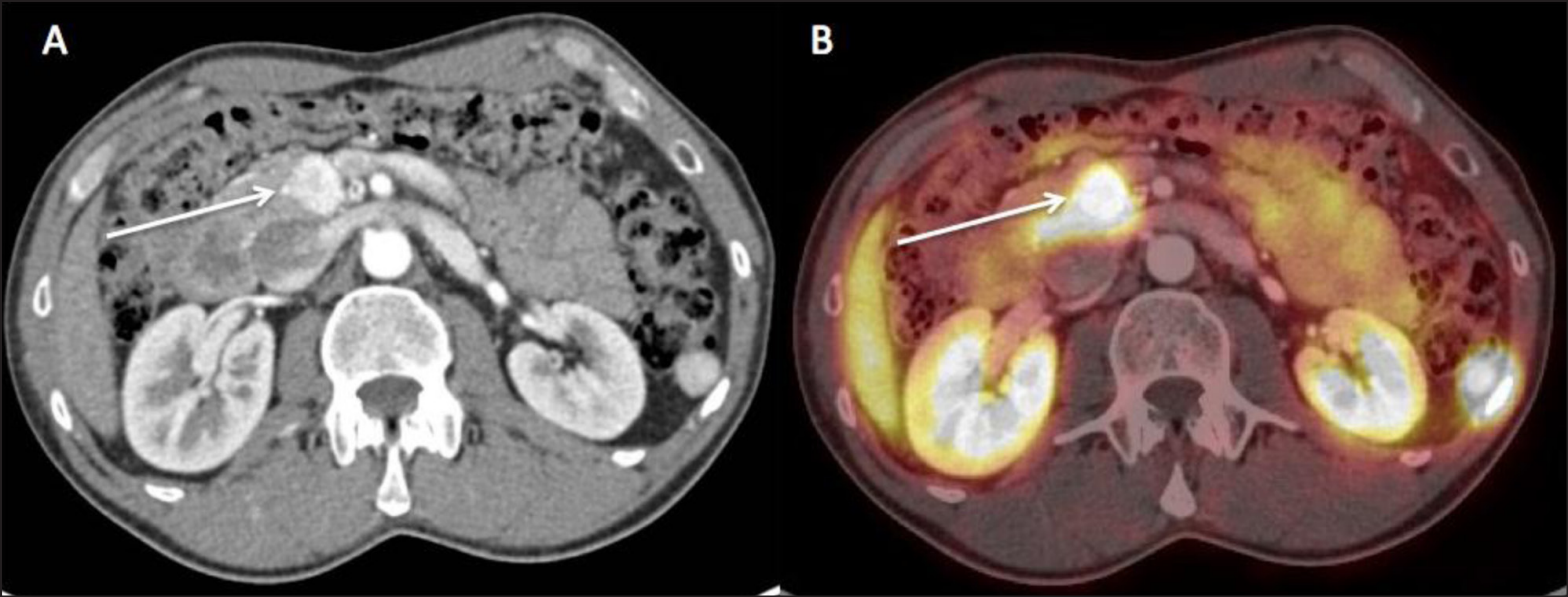


A 38-year-old man with a hypervascular mass lesion in the pancreas (Figure 1A) detected on Computed Tomography (CT) was referred to our institution for a ^68^Ga DOTATATE PET/CT to identify metastatic disease. ^68^Ga DOTATATE is an imaging agent targeting somatostatin receptors (SSTR). ^68^Ga DOTATATE PET/CT is an established method in the work-up for neuroendocrine tumors (NETs), because SSTRs are over-expressed by the majority of well-differentiated NETs.

**Figure 2:**
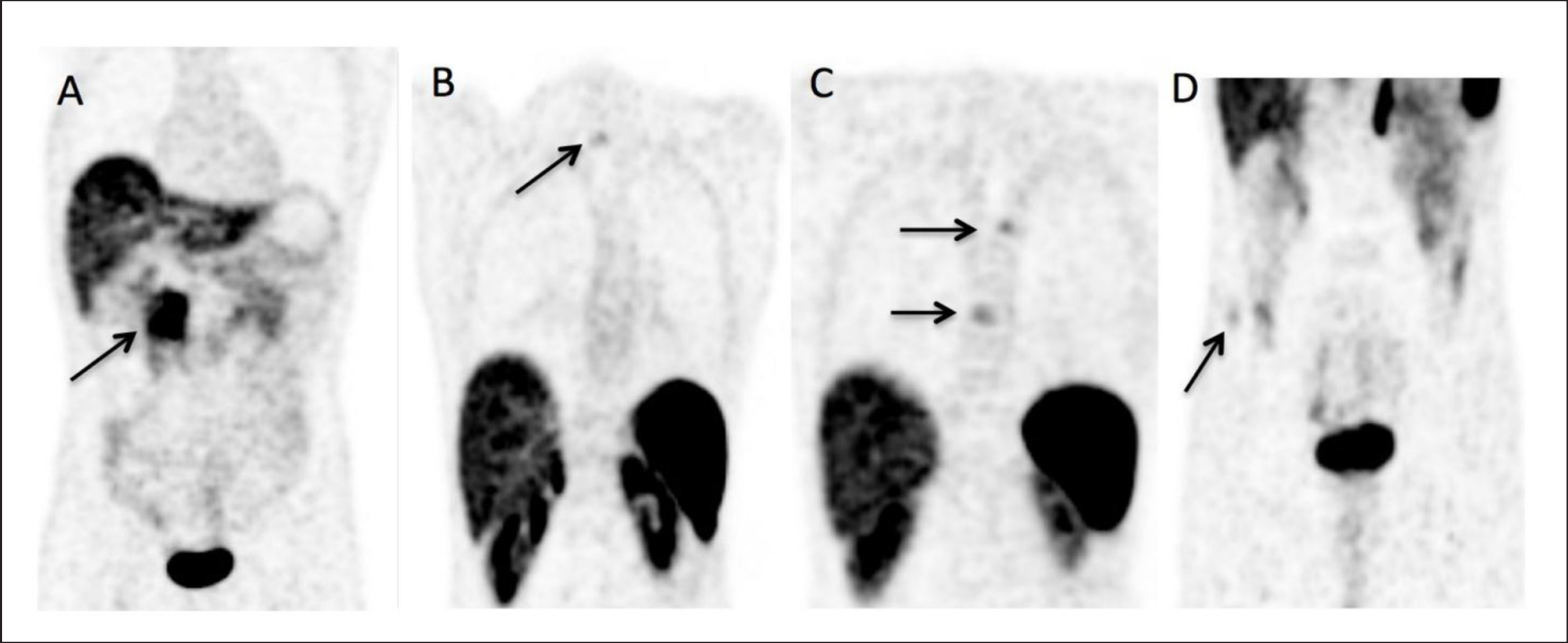


**Figure 3:**
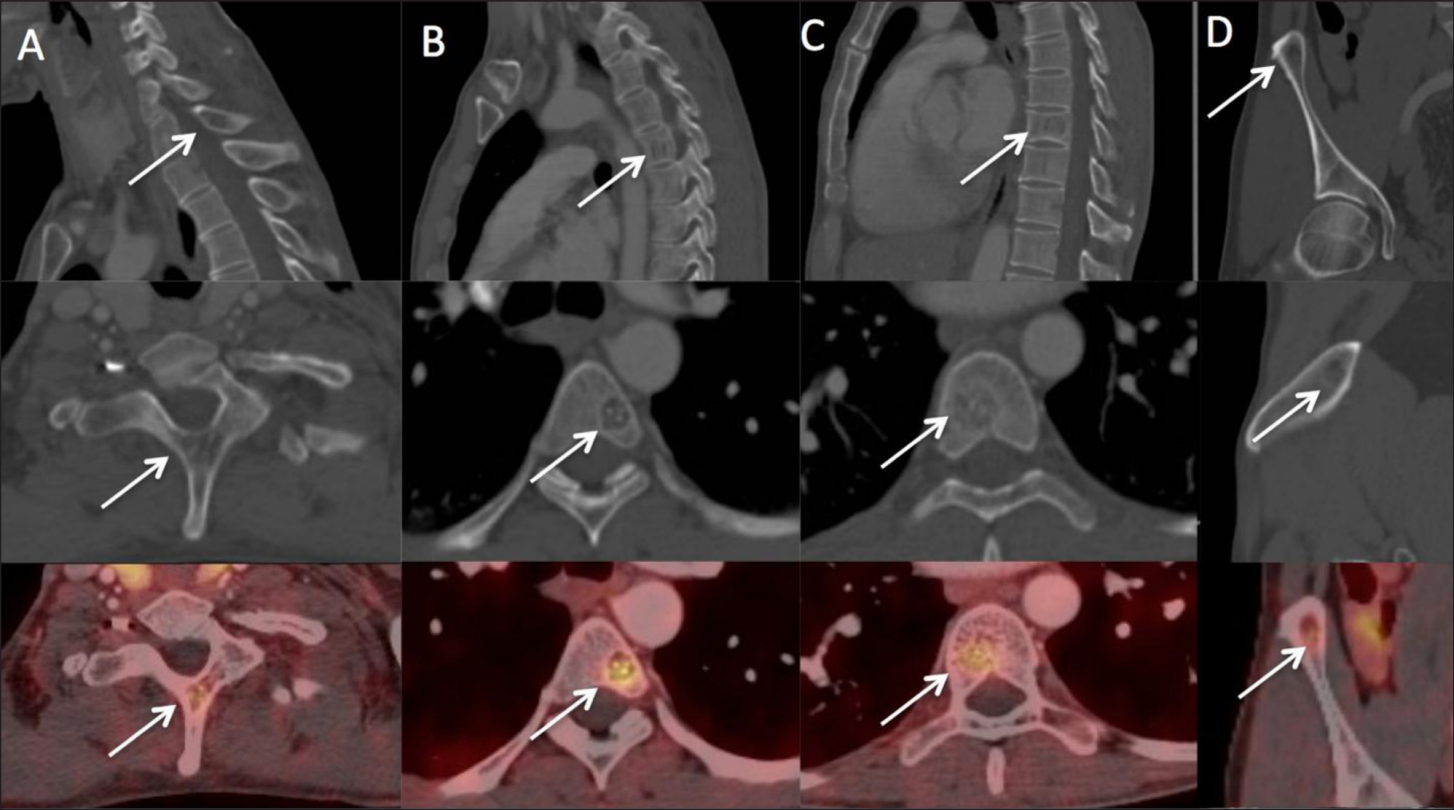


PET images showed a focus of uptake in the pancreas (Figures 1B and 2A, arrows) and moderate tracer uptake foci (arrows) in the spinous process of the first thoracic vertebra (Figures 2B and 3A), the bodies of the fifth and eighth thoracic vertebras (Figures 2C, 3B, and 3C), and the right iliac wing (Figures 2D and 3D). These uptakes of ^68^Ga DOTATATE could be taken for bone metastases. The corresponding CT images, however, showed characteristic appearances of hemangiomas, consisting in bone demineralization with vertical striation due to thickened trabeculae (“Corduroy sign” on the sagittal planes) and a “polka-dot” appearance on the axial slices where the thickened trabeculae are seen as small punctate areas of sclerosis (Figure 3, arrows).

## Comment

There are few benign differential diagnoses for the accumulation of SSTR analogs. ^68^Ga DOTATATE can accumulate in benign inflammatory diseases as activated macrophages and lymphocytes express somatostatin receptors on their surface. The exact mechanism of uptake in these benign lesions is not clear and there are very little data for hemangiomas 1.

Although bone metastasis from NETs are very rare, without evaluation of the concurrent CT images, the vertebral tracer uptake could be interpreted as skeletal metastases. Combining the anatomic and metabolic information on PET/CT is crucial for accurate tumor staging.
